# Batch sorption experiments of cesium and strontium on crushed rock and biotite for the estimation of distribution coefficients on intact crystalline rock

**DOI:** 10.1016/j.heliyon.2019.e02296

**Published:** 2019-08-13

**Authors:** Jukka Lehto, Esa Puukko, Antero Lindberg, Mikko Voutilainen

**Affiliations:** a Department of Chemistry - Radiochemistry, University of Helsinki, Finland; b Geological Survey of Finland, Espoo, Finland

**Keywords:** Electrochemistry, Crystalline rock, Cesium, Strontium, Specific surface area, Sorption, Distribution coefficient, Radionuclide

## Abstract

The distribution coefficient (K_d_) of radionuclides on bedrock is one of the key parameters used in the safety analysis of spent nuclear fuel repositories. Typically, distribution coefficients have been determined using crushed rock. However, recent studies have shown that crushing of the rock increases considerably the distribution coefficient compared with the values of intact rock. This study aimed to test if batch sorption experiments using different grain sizes (i.e. mean diameter of grains) can be used to evaluate the K_d_ of strontium (Sr) and cesium (Cs) on intact crystalline rock, which would decrease the needed experimental time compared with transport experiments. Here we report the results of the batch sorption experiments with crushed rocks and compare the results with those from a recent study performed using electromigration experiments with intact drill core samples (Puukko et al., 2018). The batch sorption experiments were done for rock samples from Olkiluoto, Finland, as a function of grain size and of Cs and Sr concentration. Furthermore, the specific surface areas of the same rock samples with different grain sizes were determined. It was shown that Cs distribution coefficients correlate with specific surface areas of the studied rocks and biotite, the correlation coefficient being 0.95. The Cs distribution coefficient was highest for biotite at about 0.1 m^3^/kg at 10^−4^ M cesium concentration and increased systematically to about 1 m^3^/kg at 10^−8^ M. Distribution coefficients for rocks were up to about two orders of magnitude lower, being lowest with the rock with the lowest biotite content (3.3%). The distribution coefficient of Sr varied from 0.04 m^3^/kg to 0.007 m^3^/kg and behaved in a different manner: it remained constant in two out of three studied rocks in the concentration range of 10^−8^-10^−4^ M and only in the case of one rock a decreasing trend was seen at the higher concentration range. It was also shown that batch sorption experiments overestimate the distribution coefficient in respect to intact rock. The decrease of the distribution coefficient as a function of grain size can be estimated using a power law function. It was also shown that estimation of distribution coefficients of Cs and Sr for intact rock by extrapolation of distribution coefficients determined for different grain sizes is not possible without increasing grain size, but in that case diffusion into the grains would also affect the results. A new method was developed for estimating the fraction of the inner surface area of the total surface area of crushed grains. For the mean grain sizes of 0.25 mm and 0.75 mm the fraction of the inner surface was found to be 35–70% and 60–90%, respectively. The inner specific surface area was highest with biotite at 1.2 m^2^/g and lowest with the rock with lowest biotite content (3.3%) at 0.07 m^2^/g. The surface area analysis revealed that crushing creates and/or allows access to additional inner surface area that is not measured in intact rock. Furthermore, it was demonstrated that sorption of Cs on crushed rock was dominated by mica minerals in multiple concentrations while the effect of mica minerals on the K_d_ of Sr was not as straightforward.

## 1 Introduction

In Finland, the spent nuclear fuel from the currently operating nuclear power reactors will be disposed in a bedrock repository at a depth of about 400 meters. In the safety assessment of the repository a long-term behavior of radionuclides in the system including man-made repository, groundwater, bedrock and biosphere has to be taken into account. In the final disposal of spent nuclear fuel in geological formations, such as bedrock in Finland, the rock above the spent fuel acts as the last barrier against the release of radionuclides into the biosphere. This has been taken into account in the safety assessment of the final disposal concept by the evaluation of the transport properties of radionuclides in the bedrock. One of the retardation mechanisms of migrating radionuclides in bedrock is sorption on the minerals (Posiva, 2013). In the safety assessment, the distribution coefficient of the radionuclide (K_d_) reflects the chemical properties of the rock and the magnitude of sorption in considered conditions (Hakanen et al., 2012). Furthermore, equilibrium and reversibility of the process are assumed for the sorption in the safety assessment. The distribution coefficients of radionuclides on bedrock depend strongly on the rock type and the mineral composition of rock, the ground water composition and the radionuclide in question.

In the past, the distribution coefficients of different minerals and rocks have been mainly determined in static batch experiments using crushed rock (Cornell, 1993; Skagius et al., 1982; Skagius and Neretnieks, 1988; Huitti et al., 1998). Crushing, however, increases the specific surface area of rock in respect of intact rock and thus the results may overestimate the distribution coefficient of intact rock. It has been found out that the distribution coefficients determined using crushed rock can be significantly higher than the ones determined using intact rock (Crawford, 2010). Previously, attempts have made to determine the distribution coefficients of intact rock using through-diffusion experiments (Tachi et al., 2011, 2015), electromigration sorption experiments (André et al., 2009a; Puukko et al., 2018), and in situ experiments (Soler et al., 2015; Voutilainen et al., 2019a). However, these experiments are time-consuming, especially for strongly sorbing nuclides such as Cs, and in the through-diffusion experiments only limited sample size can be used, which may cause problems due to sample preparation (e.g. drilling and sawing). Due to these issues it would be beneficial if batch sorption experiments could be used to determine reliable distribution coefficients for the intact rock or at least estimate them. However, it is not yet completely understood how the results of batch sorption experiments could be converted for intact rock. In safety assessment, this issue has been solved using a conversion factor for results of batch sorption experiments. Conversion factors have been obtained by comparing sorption values for crushed and intact rock samples. Another choice has been to compare their specific surface areas. In both cases determination of values for intact rock samples are laborious and time-consuming. Since data do not exist for all systems, conservative values in the safety assessment have been used for conversion factors.

In the long-term, ^135^Cs (t_½_ = 2.3 × 10^6^ a) is one of the key radionuclides that may cause a dose to humans if transported into the biosphere. Furthermore, ^90^Sr (t_½_ = 29 a) and ^137^Cs (t_½_ = 30 a), could play an important role in case of a failure of the repository during the operational stage. To these ends, it is important to study the behavior of Cs and Sr in contact with groundwater and bedrock. Cs and Sr exist as cations in the ground water solutions (Cs^+^ and Sr^2+^) (Hakanen et al., 2012; Söderlund et al., 2019) and their sorption mechanism is ion exchange (Liu et al., 2004; Cho and Sridhar Komarneni, 2009). Sorption of these nuclides on crystalline rocks has been studied widely using samples from different sites e.g. in Finland (Huitti et al., 1998; Muuri et al., 2017; Aromaa et al., 2019), Sweden (Skagius et al., 1982; Skagius and Neretnieks, 1988), Czech Republic (Videnská et al., 2015), Switzerland (Tachi et al., 2015; Muuri et al., 2016) and Japan (Tachi et al., 2011). These studies include sorption and diffusion of Cs and Sr on various rocks and minerals, including biotite, as a function of their concentration and the results have been modelled in the same way as in this paper. Tachi et al. (2011) also report on through-diffusion experiments and compare their results with batch sorption experiments. Skagius et al. (1982) is the only paper that has partly the same focus as the present one: they have studied Cs and Sr sorption on crushed granite of two grain sizes (0.10–0.12 mm and 4–5 mm). The fraction of sorption taking place on the external surface of the grains was 15–40% for the smaller grain size and only few percent for the larger. As will be later seen, these findings are well in line with ours. Our study is, however, more extensive than that of Skagius et al. (1982). Furthermore, it has been shown that pore structure and heterogeneity may play an important role in the transport of radionuclides in crystalline rock samples (Sammaljärvi et al., 2017; Kuva et al., 2018).

The bedrock of Olkiluoto consists of various gneisses and granites and their main minerals are quartz, potassium feldspar and plagioclase (Kärki and Paulamäki, 2006). Furthermore, biotite is an abundant mineral in the gneisses. Biotite, among the other mica minerals, is known to dominate the sorption capacity of the crystalline rock (Muuri et al., 2016). Previously, it has been shown that there are three different sorption sites in biotite grains, dominating the sorption process in certain concentration regions (Kyllönen et al., 2014). At low concentrations, below 10^−6^ M, sorption of Cs is dominated by frayed edge sites (FES). After the FES are occupied, the sorption is dominated by the intermediate sites. At high concentrations, above 10^−5^ M, sorption is dominated by sites on the basal planes on biotite crystal surfaces. In the case of Sr, FES do not play an important role due to their low selectivity and low number (Söderlund et al., 2019).

The main aim of this study was to test if batch sorption experiment using different grain sizes can be used to evaluate the K_d_ of intact crystalline rock samples for Sr and Cs instead of performing time-consuming diffusion and electromigration experiments. The results were compared with K_d_ determined for intact rock samples from the same locations in similar conditions (Puukko et al., 2018). The secondary aims of the study were to test how the biotite content of the different rock from Olkiluoto site affects the sorption of Cs and Sr and how the crushing of the rock samples affects the specific surface area and the distribution coefficients.

## 2 Materials and methods

### 2.1 Olkiluoto rock types and their average mineral contents

Olkiluoto bedrock consists of four major rock types (contents given in parentheses): migmatitic gneisses (ca. 64%), pegmatitic granites (ca. 20%), gneisses (ca. 9%) and tonalite-granodiorite-granite gneisses (ca. 8%) (Kärki and Paulamäki, 2006). The main minerals in these rocks are quartz (25–35%), potassium feldspar (5–35%), plagioclase (15–35%) and biotite (10–30%) (Kärki and Paulamäki, 2006). An exception for the biotite content is the pegmatitic granite where the biotite content is low (around one percent). In addition to biotite, these rocks contain other mica minerals (e.g. muscovite) but at lower content. The rocks contain also pinite and sericite, the alteration products of cordierite and plagioclase. These minerals are known to have a major effect to sorption capacity (due to their mineral structure, alteration state and chemical composition) of the rock and thus to the retention of radionuclide migration in crystalline rocks (Torstenfelt et al., 1982).

### 2.2 Rock samples

The batch sorption experiments and specific surface area measurements were performed for the two rock types that represent the typical rock types in Olkiluoto: mica gneiss (MGN) and tonalite-granodiorite-granite gneiss (TGG). The samples were selected from four different drill cores: MGN samples from drill cores PP219 and PP309 and TGG samples from drill cores PP175 and KR56. With respect to chemical composition, the TGG samples represent two subtypes, P (PP175) and T (KR56), differing in chemical composition. The average biotite contents of these two TGG subtypes in the Olkiluoto bedrock vary as well, being (22.5 ± 7.1) % for P and (8.2 ± 7.5) % for T (Kärki and Paulamäki, 2006). However, later in this report, the subdivision of the TGG rocks is not taken into account. The drill core samples for this study were provided by Posiva Oy, Finland, and they represent the typical rocks in Olkiluoto at the depth of the repository. The samples from the same drill core were used in a previous study where distribution coefficients on intact rock were determined using the electromigration sorption experiments (Puukko et al., 2018). The samples for these experiments were taken from the same drill cores as close as possible to the drill core samples for the electromigration sorption experiments. For the batch experiments the samples were first crushed and then sieved for several grain size fractions.

The batch sorption experiments were also performed using biotite, quartz, plagioclase and K-feldspar. The biotite grains were separated for these measurements from the MGN samples after crushing. The separation of all minerals was performed using wet high intensity magnetic separation followed by heavy liquid separation. Finally, the separated grains were sieved to different size fractions.

The mineral contents of the rock samples were determined using four methods: Mineral Liberation Analyzer (MLA), Field Emission Scanning Electron Microscope (FESEM), X-ray Diffractometer (XRD) and thin sections using polarization microscope and point counting method.

The MLA was done with FEI Quanta 600 with two DX-detectors (EDAX Apollo XL) and analyzed by the MLA-program using a XMOD standard method. In the analysis, an acceleration voltage of 25 kV, magnification of 132 and resolution of 512×400 pixels were used and the number of counted particles varied from 18000 to 20000 per sample.

The FESEM was performed using JEOL JSM 7100F that was combined with Inca X-sight EDS system of Oxford Instruments. The EDS signal was analyzed using software INCA Point ID and Aztec (Oxford Instruments). The FESEM was operated in the high vacuum and back scattered compo signal modes using the accelerating voltage of 20 kV and probe current of 1.3 nA.

The Bruker D8 Discover A25 device was used in XRD analyses. Samples were analyzed with an angle interval of 2-70^o^ using a measuring step of 0.02^o^ and time of 0.1 s. Voltage was 40 kV and current 40 mA; anode material was Cu. The mineral phases were identified using Eva software (Bruker) and database PDF-4 Minerals (ICDD).

The thin sections were analyzed under polarization microscope and using a point counting method (500 independent points counted per sample).

The average mineral contents of the rock samples determined using the four methods are shown in Table 1. All samples were not studied with all of the methods. The mica contents of PP175, PP309 and PP219 are typical for Olkiluoto gneisses while for KR56 exceptionally low mica content was measured. The contents of the main minerals (plagioclase, K-fledspar and quartz) are within the typical variation of Olkiluoto rocks (Kärki and Paulamäki, 2006).

**Table 1:** The average mineral contents (volume-%) of four rock samples (KR56, PP175, PP309 and PP219). The most effective sorbing minerals are highlighted.

	Average mineral content (volume %)			
Rock type	TGG		MGN	
Drill core	KR56^a^	PP175^a^	PP309^b^	PP219^b^
Sampling depth (m)	815 m	103 m	>284 m^c^	>285 m^c^
Plagioclase	36.8 ± 3.5	33.8 ± 0.8	37.9 ± 7.1	27.5 ± 6.6
K-feldspar	25.5 ± 3.6	4.7 ± 4.6	5.9 ± 4.1	5.3 ± 4.7
Quartz	33.8 ± 2.4	19.8 ± 2.1	25.8 ± 3.4	27.2 ± 4.0
**Biotite**	**3.3 ± 2.2**	**27.5 ± 2.6**	**12.0 ± 6.3**	**29.0 ± 6.4**
**Muscovite**	**0.7 ± 0.3**	**0.8 ± 0.7**	**0.3 ± 0.3**	**1.4 ± 1.2**
**Sericite**	**–**	**–**	**1.3 ± 0.3**	**1.7 ± 0.5**
**Pinite**	**–**	**–**	**–**	**1.0 ± 0.5**
Chlorite	0.10 ± 0.05	1.1 ± 0.8	15.6 ± 13.5	2.9 ± 3.6
Pyroxene	**–**	7.3 ± 0.7	**–**	**–**
Apatite	**–**	4.2 ± 1.0	**–**	**–**

a Thin section method and FESEM-EDS. Thin section results are averaged over three independent measurements.

b Thin section method, MLA and XRD. Thin section results are averaged over three independent measurements and MLA results over two.

c Starting location of drilling with dips of -89.9^○^ (PP309) and -89.5^○^ (PP219).

### 2.3 Specific surface area measurements and estimation of specific inner surface areas of the grains

For the specific surface area measurements, the rock and biotite samples were crushed and sieved in different size fractions (mean sizes were 0.10 mm, 0.20 mm, 0.375 mm, 0.75 mm and 1.5 mm). The specific surface area (SSA) was determined at the Chalmers University of Technology (Sweden) with Kr-BET using a gas adsorption analyzing instrument (ASAP 2020, Micromeritics). Prior to analysis, the samples were degassed at a pressure of about 2 μmHg for 1500 minutes at room temperature. The adsorption isotherms were evaluated for adsorbent surface area with the BET (Brunauer-Emmet-Teller) model by the instrument software (MicroActive, Micromeritics). Kr sorption gives the total specific surface area of the grains, both on the grain surfaces and in the inside of the grains excluding the pores which the aperture is smaller than the size of Kr molecules (Klobes et al., 2006). Kr cannot penetrate into the interlayer space of mica minerals, such as biotite. Since we are interested only on the surfaces capable of ion exchange, exclusion of interlayer space surface is only a positive feature since metal ions do not enter into them either. The size of Kr molecule is in the same range as the sizes of metal ion and thus the specific surface area measured by Kr adsorption gives approximately the surface area accessible for metal ions.

The specific surface area measurements were followed by an estimation of the fraction of the inner surface area of the measured total surface area. Puukko et al. (2018) have shown that the samples studied in the here have a volumetric porosity ranging from 0.1% to 0.3%. In intact samples, the interfaces of the pores and minerals of the rock form the inner surface area that is available for sorption of radionuclides (Voutilainen et al., 2019b). The specific inner surface area (S_i)_ was estimated in the following way:

1. The grains were assumed to be spheres with a mean diameter of grains (d). From now on term “grain size” is used for mean diameter of grains.
2. The density (ρ) of the grain was assumed to be 2.7 g/cm^3^.
3. The number of grains (N_g_) in each grain size fraction was determined based on the mass of the fraction.

(1) $$N_{g}=\frac{m_{tot}}{{4\pi /3\cdot \left(d/2\right)}^{3}\rho }$$

4. The outer surface area per unit mass (S_s_) was determined for different grain size fractions.

(2) $$S_{s}=4\pi {\left(\frac{d}{2}\right)}^{3}N_{g}$$

5. The total outer surface area per unit mass (S_tot_) was determined by correcting outer surface area of spheres with a roughness factor (R_F_), which is a ratio of grain surface area to that of an ideal sphere.

(3) $$S_{tot}=R_{F}S_{s}$$

6. The inner surface area per unit mass (i.e. specific inner surface area) for each grain size was determined by subtracting the total outer surface area per unit mass from the measured specific surface area (SSA).

(4) $$S_{i}=SSA-S_{tot}=SSA-R_{F}S_{S}$$

7. The R_F_ in Eq. (4) was selected so that a value giving the specific inner surface area (S_i_) per unit mass is constant for all grain size fractions (i.e. the point where the standard deviation of S_i_ finds its minimum).

The uncertainties of the determined specific inner surface areas have been determined using propagation of uncertainties for independent variables (Taylor, 1997).

### 2.4 Sorption on crushed rock and biotite

The crushed and sieved samples were used in the batch sorption experiments. Two types of experimental sets were performed with Cs and Sr. In the first set of experiments, the concentration dependence on the distribution coefficients was studied. These experiments were performed using the grain size of 0.5–1.0 mm for the rock samples and 1.0–2.0 mm for biotite and Cs/Sr concentrations from 10^−9^ M to 10^−4^ M. Stable Cs and Sr were used to adjust the desired concentration. The concentration dependence on distribution coefficients for other main minerals (quartz, potassium feldspar and plagioclase) were studied using the grain size of 0.075–0.30 mm and Cs/Sr concentrations from 10^−6^ M to 10^−4^ M.

In the second set of experiments, the grain size dependence on the distribution coefficients was studied. These experiments were performed using the average grain sizes of 0.03 mm, 0.25 mm, 0.5 mm, 0.75 mm, 1.5 mm and 3 mm for rock samples and the Cs/Sr concentrations were selected so that the equilibrium concentration would be as close as possible to the equilibrium concentrations observed in the electromigration sorption experiments using intact drill core samples (Puukko et al., 2018). The sorption experiments with various rock grain sizes were done in 10^−9^ M Cs solution and in 10^−6^ M Sr solutions. For biotite, these experiments were performed using initial Cs concentrations of 10^−6^ M, 10^−5^ M and 10^−4^ M to determine concentration dependence on the distribution coefficient with different grain sizes. The applied concentrations are within the range that could be possible in case of canister failure during the operation of the repository (Voutilainen et al., 2017).

Before the batch sorption experiments, the biotite and rocks were equilibrated in 0.01 M NaCl solution at pH of 7 for two weeks and changing the water after the first week. After this treatment the distribution coefficients of Cs and Sr were determined by batch sorption experiments using 0.5 g of solid and 20 ml of 0.01 M NaCl solution. NaCl solution was used to keep the ionic strength constant and the concentration 0.01 M was selected because the same concentration was used in the previous electromigration sorption experiments (Puukko et al., 2018). In these conditions, the speciation of Cs and Sr are Cs^+^ and Sr^2+^ (Hakanen et al., 2012; Söderlund et al., 2019). The solutions were equilibrated in 20 ml plastic vials for a week. Preliminary experiments showed that one week is sufficient to reach the equilibrium. After this, the samples were centrifuged for 20 minutes at 1000×g and filtered (0.22 μm). For the equilibrium concentration measurements, the sorption experiment solutions were traced with gamma-emitting radionuclides ^134^Cs or ^85^Sr. Their activity concentrations in the liquid phases were determined with a gamma counter (1480 Wizard, Perkin Elmer) and the distribution coefficients were determined using equation

(5) $$K_{d}=\frac{A_{0}-A}{A}\times \frac{V}{m}$$

where A_0_ is the initial activity concentration of tracer, A is the equilibrium activity concentration of the tracer, V is the total volume of the solution and m is the mass of the sample.

## 3 Results and disscussion

### 3.1 Distribution coefficients as a function of concentration

**Fig. 1:**
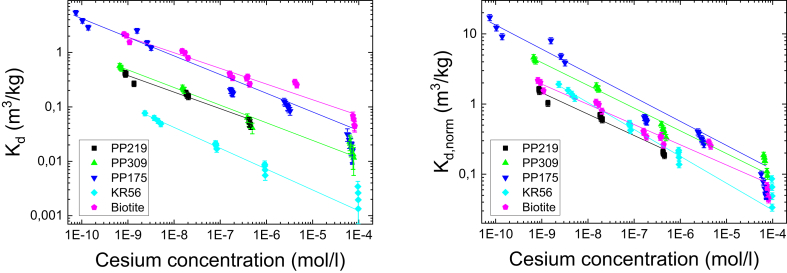
The distribution coefficient (K_d_, m^3^/kg) of Cs on crushed rocks and biotite as a function of equilibrium Cs concentration. The measured distribution coefficients (left) and normalized coefficients (K_d, norm_) according to their mica content (right) are shown. The solid lines show fits by Eq. (7). The error bars are standard deviations of the means of three parallel measurements.

Fig. 1 shows the distribution coefficient of Cs for the studied rocks (PP219, PP309, PP175 and KR56) and for biotite as a function of equilibrium Cs concentration. The measured distribution coefficients and distribution coefficients normalized to their mica content (the mean values of the sum of biotite, muscovite, sericite and pinite) are presented in the right panel of Fig. 1. The distribution coefficients decrease systematically in the concentration range studied. One would expect the distribution coefficient to be constant at lower Cs concentrations, a typical feature for sorption processes, rationalized by (Lehto and Harjula, 1999)

(6) Kd=CEC[Na]kMNa+[M]

where CEC is cation exchange capacity, [Na] the sodium ion concentration, [M] the concentration of the target ion, Cs in this case, and k_M/Na_ the selectivity coefficient (mass action quotient) for Cs to Na exchange. CEC is constant by definition and the sodium concentration in our experiments was kept constant at 0.01 M. As the term [Na]/k_M/Na_ becomes larger than [M], the distribution coefficient reaches a constant value. This was, however, not the case in our study. The distribution coefficients are still increasing at the Cs concentration of 10^−8^ M and below. This behavior indicates the very high value of a selectivity coefficient (k_M/Na_), reported being as high as 8.0 (logk_Cs/Na_) for Cs to sodium exchange in biotite (Kyllönen et al., 2014).

The data shown in Fig. 1 was fitted using a power law function

(7) $$K_{d}=a\times C^{-b}\text{,}$$

where C is the equilibrium concentration of Cs while *a* and *b* are treated fitting parameters. As the results of the analysis it can be seen that there is a good agreement with measured data sets and power law. The values for parameter *b* vary from 0.29 to 0.38 in both cases as the normalization does not affect parameter *b*. The pure biotite has the smallest value for parameter *b* which indicates that other minerals affect only slightly the results of crushed rock. The difference between distribution coefficient values with these rocks is somewhat more than 10-fold range. For example, at 10^−6^ M Cs concentration the largest difference is 14-fold.

When the distribution coefficients are normalized to the mica mineral contents of the rocks, the distribution coefficients become considerably closer to each other (see Fig. 1, right panel). In this case, the largest difference between various rocks is only 2.7-fold at 10^−6^ M Cs concentration. Moreover, the distribution coefficient of Cs on biotite falls in the range observed for rocks. These findings indicate that biotite, together with other mica minerals, is the major component responsible for Cs sorption in the studied rocks. From the main minerals quartz, potassium feldspar and plagioclase the two first did not sorb practically any Cs from the solution at the studied concentration range of 10^−6^ M−10^−4^ M while plagioclase sorbed Cs rather efficiently, the K_d_ being in this range on average 0.044 ± 0.028 m^3^/kg which is 19% of the corresponding value for biotite. The rather high sorption capacity of plagioclase can be explained by seritization and sausseritization of plagioclase that has occurred in Olkiluoto bedrock in some extent (Aaltonen et al., 2010).

In general, the results in Fig. 1 show strong non-linear concentration dependence on the sorption isotherm. However, in the current safety assessments calculations constant-K_d_ models with various realizations have been used (Posiva, 2013). The observations presented here are in fair agreement with the results of previous studies on crystalline rock (Muuri et al., 2016, 2017). In the case of granodiorite and pegmatite Muuri et al. (2016, 2017) observed a similar trend, increase with decrease in Cs concentration, as in our study but in the case of biotite and veined gneiss they observed a constant K_d_ at Cs concentration at 10^−7^ M and below.

**Fig. 2:**
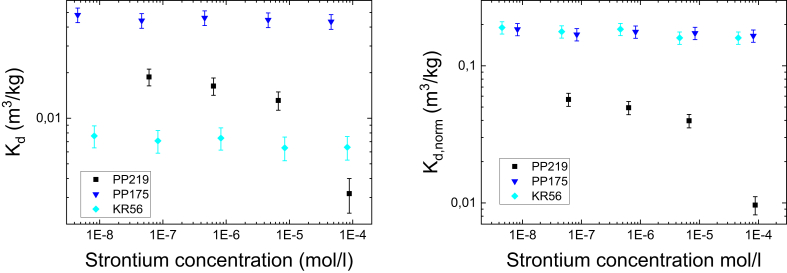
The distribution coefficient (K_d_, m^3^/kg) of Sr on crushed rocks a function of equilibrium Sr concentration. The measured distribution coefficients (left) and normalized coefficients according to their mica content (right) are shown. The error bars are standard deviations of the means of three parallel measurements.

Fig. 2 shows the distribution coefficient of Sr from batch sorption experiments for the studied rocks (PP219, PP175 and KR56) as a function of equilibrium Sr concentration. The dependence of the distribution coefficient on the Sr concentration was very different from that of Cs. While the Cs distribution coefficient shows a decreasing trend with increasing Cs concentration in the whole concentration range studied (10^−10^-10^−4^ M), the distribution coefficient of Sr remains fairly constant in the concentration range from 10^−8^ M to 10^−5^ M for the rocks KR56 and PP175 and only in case of PP219 the distribution coefficient starts to decrease at the Sr concentration of about 10^−5^ M. Normalization of the K_d_ values to the mica contents gave more or less identical K_d_ values between PP175 and KR56, while PP219 differed considerably. Based on these results, conclusions regarding dependence of the distribution coefficient and abundance of mica minerals cannot be drawn and it is evident that other properties of the rock (e.g. abundance of other reactive minerals) may have a strong influence on the distribution coefficient of Sr.

### 3.2 Distribution coefficient as a function of grain size

**Fig. 3:**
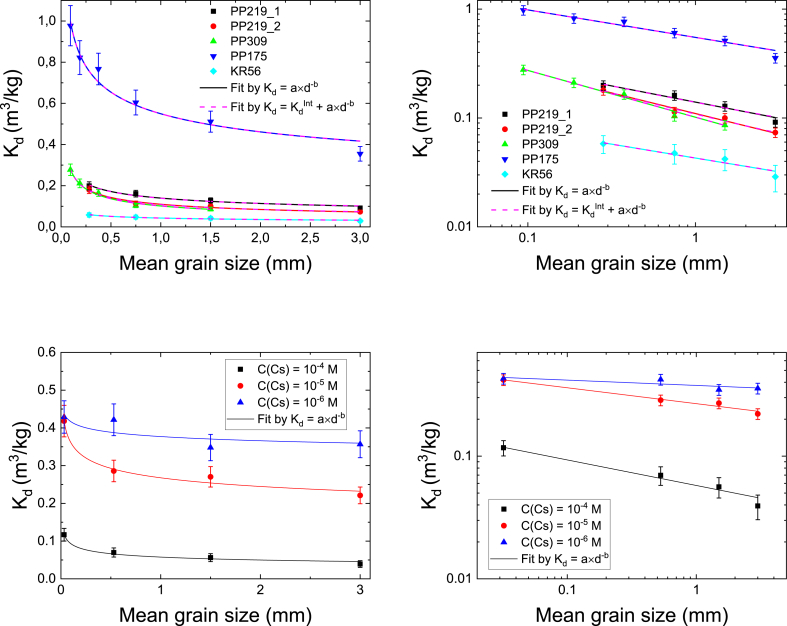
The distribution coefficient of Cs on PP219_1, PP219_2, PP309, PP175 and KR56 (upper panels) and on biotite with different Cs concentrations (lower panels) as a function of mean grain size. The graphs are shown on a linear scale (left) and on a logarithmic scale (right). Solid lines show fits by Eq. (8) and dashed lines by Eq. (9). The values for fitting parameters a an b are given in Table 2. The experiments for crushed rock samples have been performed in Cs concentration of 1×10^−9^ M. The error bars are standard deviations of the means of three parallel measurements.

The distribution coefficient of Cs as a function of grain size was determined for biotite and two rock types MGN (drill cores PP219 and 309) and TGG (PP175 and KR56). Two parallel sets of PP219 were explored since it was seen that PP219_1 contained biotite in veins while biotite in PP219_2 was in grains. It is evident that crushing of the intact rock creates additional surfaces for sorption to take place. The set of experiments was performed to find out if the batch sorption experiments using different grain sizes can be used to extrapolate reliably the K_d_ values for an infinite grain size (i.e. intact rock). Here it is assumed that the sorption capacity inside the mineral grains remains the same while the ion uptake per unit mass on the outer surface of the grains decreases with increasing grain size. The distribution coefficient of all studied rocks and biotite for Cs are shown in Fig. 3 as a function of grain size. Separate graphs show the same data on a linear scale and on a logarithmic scale fitted with a power law function

(8) $$K_{d}=a\times d^{-b}\text{,}$$

where *a* and *b* are fitting parameters. Furthermore, for studied rock the experimental curves are fitted with equation

(9) $$K_{d}=K_{d}^{int}+a\times d^{-b}\text{,}$$

where $K_{d}^{int}$ is the K_d_ of intact rock that was determined for each rock type by Puukko et al. (2018). When data is fitted using Eq. (9), $K_{d}^{int}$ is an asymptotic value of K_d_ when GS approaches infinity, i.e. K_d_ for intact rock.

The fits by different equations show that $K_{d}^{int}$ does not affect the fit at the studied grain sizes and that in this scale simple power law can be used to describe the decrease of K_d_ as a function of grain size. It can be assumed that much larger grain sizes would be needed to obtain $K_{d}^{int}$. However, in such experiments the effect of the effective diffusion coefficient has to be taken into account meaning that the experimental and analysis requirements are increased and the simplicity of the batch sorption experiments are lost (Muuri et al., 2018). Furthermore, if the sorption would take place only on the outer surfaces of the grains, the distribution coefficient would decrease as a function of grain size so that the constant *b* in Eq. (8) would be 1 (André et al., 2009b). This is evidently not the case and the decrease is not as dramatic and values for *b* vary from 0.25 to 0.43 (see Table 2). This indicates that there are inner surfaces in grains capable for sorption. The fraction of the inner surface area of the total surface area is discussed below. Two PP219 samples, containing biotite in a different manner, behaved in a more or less identical way. The mica mineral content of the samples seems to affect considerably the distribution coefficients in all grain sizes as it was shown in the previous section using one grain size.

**Table 2:** Fitting parameters a and b for Cs fits by Eqs. (8) and (9) used in the plots shown in Fig. 3. The values for $K_{d}^{int}$are from Puukko et al. (2018).

	Fitted Eq.	$K_{d}^{int}$ (m^3^/kg)	a (m^3^/kg)	b (-)
PP219_1	Eq. (8)	–	0.140 ± 0.006	0.30 ± 0.05
	Eq. (9)	0.02	0.119 ± 0.007	0.34 ± 0.06
PP219_2	Eq. (8)	–	0.110 ± 0.005	0.38 ± 0.04
	Eq. (9)	0.02	0.089 ± 0.004	0.46 ± 0.05
PP309	Eq. (8)	–	0.043 ± 0.002	0.25 ± 0.05
	Eq. (9)	0.0027	0.040 ± 0.002	0.27 ± 0.06
PP175	Eq. (8)	–	0.60 ± 0.03	0.25 ± 0.03
	Eq. (9)	0.0045	0.55 ± 0.03	0.25 ± 0.03
KR56	Eq. (8)	–	0.102 ± 0.006	0.43 ± 0.03
	Eq. (9)	0.0007	0.101 ± 0.006	0.43 ± 0.03
Biotite, 1×10^−4^ M	Eq. (8)	–	0.058 ± 0.004	0.21 ± 0.03
Biotite, 1×10^−5^ M	Eq. (8)	–	0.268 ± 0.009	0.130 ± 0.013
Biotite, 1×10^−6^ M	Eq. (8)	–	0.38 ± 0.02	0.043 ± 0.03

The graphs for the distribution coefficient of Cs on biotite as a function of grain size are shown with Cs concentrations of 10^−6^ M, 10^−5^ M and 10^−4^ M (see Fig. 3). It is not possible to determine a $K_{d}^{int}$ for biotite and thus only fit Eq. (8) is presented. The simple power law describes relatively well the behavior of K_d_ as a function of grain size and the K_d_ decreases in all concentrations with the exponent from 0.043 to 0.21 (see Table 2). The decrease of K_d_ can be explained by the decrease of specific surface area as a function of grain size which is further discussed below. In the studied grain size range, the relative decrease is the strongest in the highest concentration and weakest in the lowest concentration. Furthermore, it can be concluded that distribution coefficient of Cs decreases as a function of Cs concentration with all studied grain sizes. Even though the experiments of rocks were done at a different concentration than those of biotite we may assume, based on the values for fitting parameters *a* and *b* (see Table 2), that grain size affects less the distribution coefficient of biotite than that of crushed rock. This indicates that the fraction of inner surface area of the total surface is higher for biotite than for the rock. This is discussed in more detail below. Note also that results for the distribution coefficients of the rocks and biotite cannot be directly compared since the experiments have been performed in different concentrations. In the concentration of 1×10^−9^ M, the sorption on biotite is dominated by the FES while in the concentration range of 1×10^−6^–1×10^−4^ basal sites are dominating the sorption (Kyllönen et al., 2014).

**Fig. 4:**
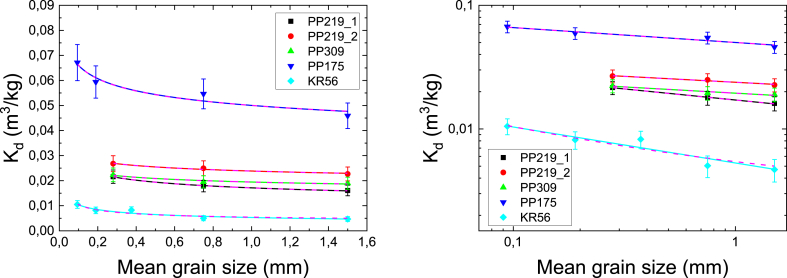
The distribution coefficient of Sr on PP219_1, PP219_2, PP309, PP175 and KR56 as a function of mean grain size. The graph is shown on a linear scale (left) and on a logarithmic scale (right). Solid lines show fits by Eq. (8) and dashed lines by Eq. (9). The experiments have been performed in Sr concentration of 1×10^−6^ M. The error bars are standard deviations of the means of three parallel measurements.

The distribution coefficients of Sr on the crushed rocks as a function of grain size behave similarly as those of Cs (see Fig. 4). The same power law (see Eq. (8)) can be used to explain the behavior of the distribution coefficient within the studied grain sizes. Furthermore, the fits by Eq. (9) do not change considerably from the ones by Eq. (8) when using the values reported by Puukko et al. (2018) for the distribution coefficient of intact rock ($K_{d}^{int}$) (see Table 3). This indicates that the results of batch sorption experiments cannot be extrapolated in order to estimate the distribution coefficient of Sr in intact rock. In general, the distribution coefficients of Sr are smaller (see Figs. 1 and 2) and the effect of grain size is milder than in the case of cesium.

**Table 3:** Fitting parameters a and b for Sr fits by Eqs. (8) and (9) used in the plots shown in Fig. 4. The values for $K_{d}^{int}$are fixed according Puukko et al. (2018).

	Fitted Eq.	$K_{d}^{int}$ (m^3^/kg)	a (m^3^/kg)	b (-)
PP219_1	Eq. (8)	–	0.0171 ± 0.0002	0.179 ± 0.013
	Eq. (9)	0.0044	0.0127 ± 0.0002	0.235 ± 0.014
PP219_2	Eq. (8)	–	0.0239 ± 0.0004	0.10 ± 0.02
	Eq. (9)	0.0044	0.0195 ± 0.0004	0.12 ± 0.03
PP309	Eq. (8)	–	0.0195 ± 0.0005	0.10 ± 0.03
	Eq. (9)	0.0044	0.0151 ± 0.0005	0.13 ± 0.04
PP175	Eq. (8)	–	0.050 ± 0.002	0.12 ± 0.03
	Eq. (9)	0.0073	0.043 ± 0.002	0.14 ± 0.03
KR56	Eq. (8)	–	0.0053 ± 0.0006	0.29 ± 0.06
	Eq. (9)	0.003	0.0024 ± 0.0006	0.48 ± 0.12

Based on these experiments it can be concluded that batch sorption experiments with grain size smaller than 3 mm overestimate the K_d_ of intact rock and that batch sorption experiments cannot be used to estimate K_d_ of intact rock by using a simple approach by Eq. (9). Clearly, further methods are needed and they have been discussed in recent work by Puukko et al. (2018) where the electromigration sorption experiments were used to determine the distribution coefficients of intact rock.

### 3.3 Specific surface area as a function of grain size

**Fig. 5:**
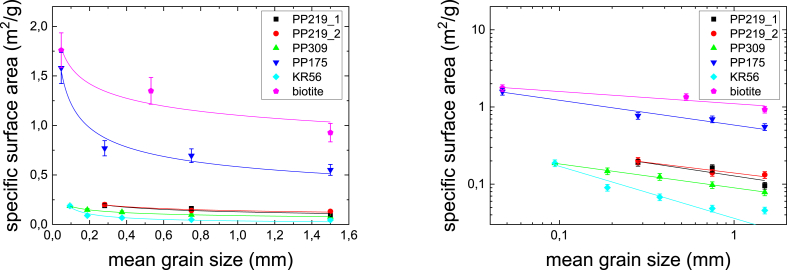
The specific surface areas (m^2^/g) of studied rocks (PP219_1, PP219_2, PP309, PP175 and KR56) and biotite as a function of grain size. The results presented in linear (left) and logarithmic scale (right) and solid lines show fits by Eq. (8).

The specific surface areas were measured for three to five different grain sizes of crushed rocks (PP219_1, PP219_2, 309, PP175 and KR56) and biotite. The results are given in Fig. 5 using linear and logarithmic scales. The results are fitted using a power law function, similar to Eq. (8) (replacing K_d_ with specific surface area). If the specific surface area originated only from the outer surfaces of the grains, the value for parameter *b* in Eq. (8) would be 1 (André et al., 2009b). This, however, is not the case and the fitted values of parameter *b* vary from 0.16 to 0.67. This reveals that the grains are porous and have also inner surfaces. This is what was expected and in the treatment given in Section 2.3 is applied to estimate the fraction of the inner surface area of the total surface area.

**Fig. 6:**
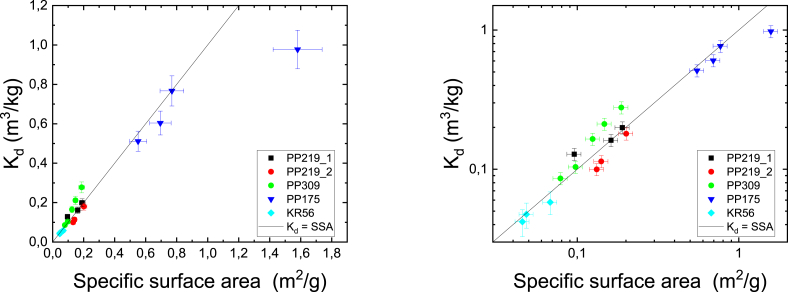
The distribution coefficients (K_d_, m^3^/kg) of Cs for studied rocks (PP219_1, PP219_2, PP309, PP175 and KR56) as a function of specific surface area (SSA, m^2^/g). The results presented in linear (left) and logarithmic scale (right). Solid line shows trend line K_d_ (m^3^/kg) = SSA (m^2^/g) and correlation coefficient of K_d_ of Cs and SSA is 0.95. The different data points for each rock sample correspond to different grain sizes.

The distribution coefficient of Cs for studied rocks as a function of the specific surface area is shown in Fig. 6 using linear and logarithmic scales. The distribution coefficients of Cs correlate strongly with the specific surface area. The distribution coefficient of Cs increases nearly linearly with increasing specific surface area for all studied rock samples. The determined data points are mostly close to the trend line K_d_ (m^3^/kg) = SSA (m^2^/g). At Cs concentration of 10^−6^ M the correlation coefficient between the distribution coefficients and the specific surface areas is 0.95 (the correlation is statistically significant as the two-tailed probability factor is clearly below 0.05 at 0.013).

**Fig. 7:**
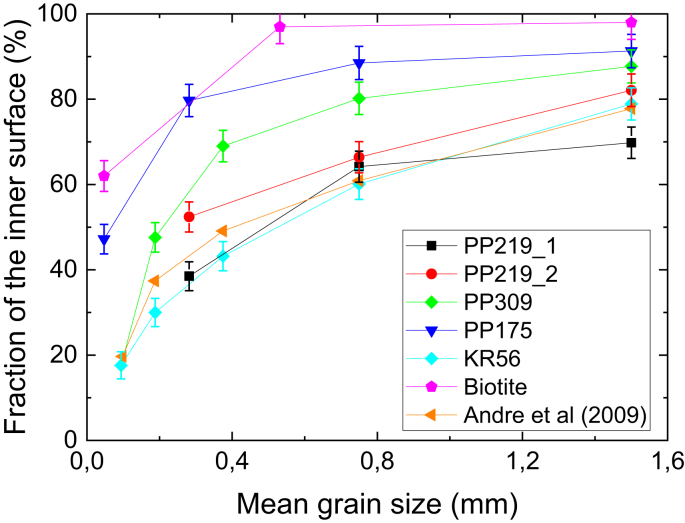
The fraction of inner surface area of the measured specific surface area for different rock samples and biotite as a function of average grain size. The error bars represent the uncertainty of the approach given in 2.3. The results of similar analysis by André et al. (2009b) are also given. Note that the solid lines are used only to connect the data points.

The outcome of the inner specific surface area analysis is shown in Table 4. The estimated inner specific surface area is the highest for biotite and varies considerably from one rock sample to another. For the rock samples, the inner specific surface area correlates moderately with the distribution coefficient (correlation coefficient 0.67): The inner specific surface areas and distribution coefficients are the highest for PP175 and lowest for KR56 (see Figs. 3 and 4) that has the lowest mica mineral content. Whereas both values for samples PP309 and PP219 are similar. The roughness factor determined using the approach in Sec. 2.3 varies in the range from 11 to 33. As Fig. 7 shows, the outer surface area dominated only in the smallest grains sizes and for grain sizes higher than 0.8 mm the most of the surface area compose of the inner surface area. In biotite and sample PP175, over 80% of the specific surface area consist of the inner surface area for grain sizes larger than 0.3 mm.

**Table 4:** The inner specific surface area and roughness factor for rock samples and biotite determined using the approach given in Sec. 2.3. Note that according the assumption made in Sec. 2.3 these values do not depend on grain size.

	Inner specific surface area (m^2^/g)	Roughness factor (-)
PP219_1	0.08 ± 0.02	33
PP219_2	0.102 ± 0.008	27
PP309	0.07 ± 0.02	11
PP175	0.7 ± 0.3	32
KR56	0.028 ± 0.008	11
Biotite	1.2 ± 0.2	24

With respect to the major aim of this study, gaining understanding for sorption parameters and specific surface area determined for crushed rock, we can conclude from these findings that crushing creates new surface area that is capable of sorbing radionuclides. As it was shown in Figs. 3 and 4, the distribution coefficients for intact rock cannot be determined based on the batch sorption experiments. However, the analysis shown in Fig. 7 indicates that with grain sizes larger than 0.8 mm the most of the surface area lies within the inner surfaces of the grains. These findings are in contradiction to each other since with the largest grain sizes most of the available surface is within inner surfaces but the distribution coefficients are still far away from the ones of intact rock. This can only be explained by creation of new inner surface area due to the crushing process. The disagreement is the largest for biotite grains that are known to be brittle and thus prone to the creation of new inner surface area. This fact makes it even more difficult to determine the distribution coefficient of intact rock based on batch sorption experiments as it is impossible to estimate the specific inner surface area created by the crushing process.

André et al. (2009b) have done a similar analysis on the rocks from Swedish nuclear fuel disposal study sites. They determined the specific surface areas of nine drill core samples with a diameter of 50 mm and length of 100 mm. In addition, they determined the specific surface of each drill core crushed to five different grain sizes of approximately the same size as in our study. We reanalyzed their data with our methodology and observed a similar trend in the fraction of inner surface area as function of grain size (see Fig. 7). Furthermore, determining the specific surface area for intact rock by fitting the surface area versus the grain size with an exponential function gave approximately the same surface areas for intact rock as the surface area versus inverse of the grain size method, on average our values for intact rock were 1.3-times higher than those of André et al. (2009b).

## 4 Conclusions

Based on the batch sorption experiments performed for multiple Olkiluoto rock samples at different grain sizes we found out that the results of batch sorption experiments cannot be directly extrapolated to infinite grain size (i.e. intact rock) by using a reasonable range of grain sizes. Furthermore, we reported a new method for estimating the fraction of inner surface area of the total surface area of the crushed grains. The analysis revealed that most of the specific surface area lies within the inner surfaces of the grains. However, it also revealed that the specific inner surface area was increased due to the crushing of the rock, which makes the conversion even more complicated. Furthermore, it was demonstrated that distribution coefficients of Cs for crystalline rock are directly proportional with mica mineral content and specific surface area. However, the heterogeneity of the rock and alteration state of the minerals may play an important role. The effect of abundance of mica minerals on the K_d_ of Sr was not as straightforward. Evidently, further attempts for finding a realistic conversion of distribution coefficients from batch sorption experiments to intact rock are needed.

## Declarations

### Author contribution statement

Mikko Voutilainen, Jukka Lehto: Conceived and designed the experiments; Analyzed and interpreted the data; Wrote the paper.

Esa Puukko, Antero Lindberg: Performed the experiments; Contributed reagents, materials, analysis tools or data.

### Funding statement

This work was supported by Posiva Oy, Olkiluoto, Finland.

### Competing interest statement

The authors declare no conflict of interest.

### Additional information

No additional information is available for this paper.
